# 

**Published:** 1888-02

**Authors:** 


### The Independent Practitioner—Vol. IX.

### PUBLISHED BY DENTISTS FOR DENTISTS.

### Prospectus.

THE INDEPENDENT PRACTITIONER, from the commencement, has constantly increased in circulation and influence. We propose to make Vol. IX better than any of the previous volumes. For the purpose of bringing it more prominently before dentists, and at the same time to place in their hands one of the most valuable books of reference yet published, THE INDEPENDENT PRACTITIONER has made arrangements with Prof. C. H. Stowell, of Ann Arbor, Mich., which enables it to offer his "Microscopic Structure of a Human Tooth," in connection with subscriptions, at an unprecedentedly low rate. This magnificent work, which is published in the form of an atlas containing twelve plates, each twelve by sixteen and one-half inches in size, undoubtedly enables the student and practitioner to get a clearer and more comprehensive idea of the various tissues of the human tooth than can be obtained by any other means. The accompanying text fully explains the illustrations, and gives a concise and clear account of the histological structure of the human tooth. The plates are printed on heavy board, the paper and press-work are like those of some holiday annual, while the cover of the portfolio is in very beautiful imitation of alligator skin. The whole forms a handsome and very appropriate ornament for the office table. Any dentist who desires to obtain a clear and accurate idea of the appearance of the intimate dental tissues, or who desires to impart this information to others, should possess this work.

It was published at the moderate price of SIX DOLLARS, and Prof. Stowell assures us that after this announcement it will not be sold singly for less. We have made such arrangements that we can furnish it in connection with a subscription to **The Independent Practitioner for One Dollar and Fifty Cents. That is, to every one who will send in Four Dollars we will send the Independent Practitioner one year and a copy of this beautiful and useful atlas.**

This offer is intended only for subscribers in America. Foreign residents within the postal union must enclose fifty cents more for prepayment of postage upon the Journal. Our regular rate of subscription to such are three dollars per annum.

As the book absolutely costs more than the sum for which it will be furnished, the money must accompany the subscription. Nor will the book be sold separately. It will only be sent in connection with a subscription to this journal.

The book will be sent either by mail or express. If it is desired that it should be sent by mail, the postage (twenty-five cents) should be sent. All remittances should be made to the Editor, Dr. W. C. Barrett, No. 208 Franklin Street, Buffalo, N. Y. Postal Orders, Postal Notes, or New York Drafts are the safest and most convenient.

| | | |
|---|---|---|
| ABBOTT FRANK...N. Y. | CARR, WILLIAM,....New York. | HILL, O. E.......Brooklyn. |
| BARRETT, W. C. Buffalo. | DUDLEY, A. M......Salem, Mass. | MILLER, W. D. Berlin, Ger. |
| BODECKER, C. F.W. N.Y. | FRANCIS, C. E.........New York. | PALMER, S. B...Syracuse. |

